# Clinical outcomes of frozen–thawed single blastocyst transfer derived from low-quality day 3 embryos: A retrospective cohort study

**DOI:** 10.3389/fendo.2025.1583779

**Published:** 2025-07-28

**Authors:** Xinyan Zhao, Qiongge Zhou, Yichun Guan

**Affiliations:** ^1^ Department of Reproductive Medical Center, Third Affiliated Hospital of Zhengzhou University, Zhengzhou, China; ^2^ The Second Clinical Medicine college, Henan University of Chinese Medical, Zhengzhou, China

**Keywords:** low-quality embryos, morphological score, frozen-thawed transfer, single blastocyst, clinical outcomes

## Abstract

**Purpose:**

Our aim was to explore the clinical outcomes of a single blastocyst frozen–thawed transfer (single blastocyst frozen–thawed transfer (singleton frozen embryo transfer, sFET) derived from low-quality day 3 (D3) embryos.

**Methods:**

This retrospective cohort study was conducted at the Reproductive Health Center of the Third Affiliated Hospital of Zhengzhou University. All data on sFET were collected between March 2016 and September 2022. Blastocysts derived from good-quality and low-quality D3 embryos were designated as the good-quality group and the low-quality group, respectively. Patients were divided into three groups according to age: <35 group, 35–39 group, and ≥40 group. Based on whether preimplantation genetic testing (PGT) was performed or not, the blastocysts derived from low-quality embryos were divided into the PGT group and the non-PGT group, respectively.

**Results:**

After adjusting for female age, male age, infertility duration, and other potential confounders, the difference in the clinical pregnancy rate and the live birth rate in the good quality and low-quality groups maintained statistical significance [adjusted odds ratio adjusted odds ratio (aOR) = 0.32 and 0.35, *p* < 0.001]. When adjusting for embryo quality, the clinical pregnancy rate and the live birth rate in the <35 and 35–39 groups were significantly higher than those in the ≥40 group (OR = 3.02 and 3.56, *p* < 0.001; OR = 1.89 and 1.84, *p* < 0.001). Embryo quality significantly affected the clinical pregnancy rate and the live birth rate (*p* < 0.001). The clinical pregnancy rate and the live birth rate in the PGT group were higher than those in the non-PGT group (40.0% *vs*. 29.3% and 40.0% *vs*. 22.0%, respectively).

**Conclusion:**

D3 embryos with low score/low quality can still obtain a certain live birth rate after further culturing to blastocysts with PGT.

## Introduction

In the process of *in vitro* fertilization embryo transfer (IVF-ET) treatment, the majority of reproductive centers select high-quality or high-morphological-score embryos for transplantation or freezing after performing morphological scoring on the third day (D3) after fertilization. Low-quality embryos with poor developmental potential would be discarded after informed consent. However, it is still controversial whether low-quality D3 embryos have clinical value or not (1). Emerging evidence indicates that vitrified–thawed blastocysts originating from poor-quality D3 embryos are capable of establishing viable pregnancies and delivering healthy offspring (2, 3). Stecher et al. demonstrated that culturing low-quality D3 embryos to blastocysts prior to vitrification could improve the utilization rate of embryos and the cumulative pregnancy rate of cycles (4). The above studies indicate that even low-quality D3 embryos may still show better developmental potential during blastocyst culture.

Studies have shown that a large proportion of embryos with high morphological scores may be aneuploid, while some low-quality D3 embryos may also be euploid (5, 6). However, in the majority of cases, the correlations between aneuploidy and the morphologies of embryos have been weak (5).

Clinically, due to advanced age, decreased ovarian reserve, and other reasons, some patients do not have high-quality embryos. For these patients, the use of embryos with low quality and poor development potential will be of great significance. This study aimed to explore the clinical pregnancy outcomes of a single blastocyst frozen–thawed transfer derived from low-quality D3 embryos.

## Materials and methods

### Participants

This study was conducted at the Reproductive Health Center of the Third Affiliated Hospital of Zhengzhou University. All data on single blastocyst frozen–thawed transfer (singleton frozen embryo transfer, sFET) were collected between March 2016 and September 2022.

The inclusion criteria were as follows: 1) infertile couples who had experienced IVF-ET due to female tubal factors and male factors, among others, and 2) sFET. The exclusion criteria were as follows: 1) patients who donated sperm or oocytes; 2) patients with incomplete medical records; and 3) patients who had experienced recurrent implantation failure.

All sFETs derived from low-quality D3 embryos were divided into the pre-implantation genetic testing (PGT) group and the non-PGT group based on whether PGT was performed.

### Methods

#### Ovarian stimulation program

Based on the woman’s age and ovarian reserve function, the clinician would formulate an appropriate scheme for ovulation promotion (7). Oocyte retrieval was performed under ultrasound guidance at 36–38 h after human chorionic gonadotropin (hCG) injection.

#### *In vitro* fertility and embryo culture

Based on oocyte maturity and sperm quality on the day of oocyte retrieval, the oocytes were inseminated via *in vitro* fertilization (IVF) or intracytoplasmic sperm injection (ICSI) at 38–40 h after hCG injection. After 16–18 h, the appearance of two evident pronuclei indicates fertility. The zygotes were cultured in the cleavage medium (G-1 PLUS; Vitrolife, Gothenburg, Sweden).

The embryos were transferred from the cleavage medium into the blastocyst medium (G-2; Vitrolife, Gothenburg, Sweden) on day 3 after insemination for development into blastocysts. Subsequently, they were cultured until day 7 after insemination in humidified air maintained at 37°C under a 6% CO_2_ and 5% O_2_ atmosphere. The development of blastocysts was observed and scored during this period.

In non-PGT treatment cycles, the blastocysts were either transferred in the fresh cycle or cryopreserved for subsequent FET based on the clinical indications and patient-specific factors. For the PGT cycles, once the blastocysts had developed, three to five trophoblast cells were taken for genetic testing, and then the blastocysts were cryopreserved. The decision to perform the transplantation.

#### Embryo score

D3 embryos were scored according to the following criteria: grade I—blastomere number (BL) of 6–10, of equal size, and fragmentation (FR) = 0%-5%; grade II—BL = 6–10, slightly equal in size, and FR = 5%–24%; grade III—BL = 6–10, unequal in size, and FR = 25%–49% or BL = 4–5 or >10; and grade IV—severely unequal-sized blastomeres, or FR > 50%, or embryo arrest. Grades I, II, and III indicate good-quality embryos, of which grades I and II are top-quality, and grade IV indicates low-quality embryos.

In our center, dependent on the situation of the patients, one or two good-quality embryos on D3 were chosen for freezing or transfer, while the others were cultured and frozen when they developed into blastocysts.

The blastocysts were observed and scored according to Gardner (8) on D5, D6, and D7 after insemination. Blastocysts at stage 3 or higher with an inner cell mass (ICM) score ≥B were considered for transfer or freezing. Blastocysts that scored 4BB or higher were considered top-quality blastocysts. Blastocysts derived from good-quality D3 embryos and bad-quality D3 embryos were defined as the good-quality group and the low-quality group, respectively.

#### Vitrification and warming of blastocysts

Vitrification and warming of the blastocysts were carried out according to the instructions in the Vit Kit (Kitazato Biopharma, Shizuoka, Japan). Before vitrification, the Vit Kits were stored at room temperature for at least 30 min. First, the blastocysts were incubated for 10 min in an equilibration solution, followed by a vitrification solution for 60 s. Subsequently, the blastocysts were placed in a carrier before being loaded into a cannula in liquid nitrogen. During warming, the cannula was taken off, the carrier end was rapidly immersed in a thawing solution (TS) at 37°C, and the blastocyst was kept there for 1 min. Then, the blastocyst was transferred to a diluent solution for 3 min, followed by washing solutions 1 and 2 for 3 min. Finally, the blastocyst was placed in blastocyst medium (G-2; Vitrolife, Gothenburg, Sweden) for transfer.

#### Endometrial preparation

The routine scheme of our center (9) was adopted for the endometrial preparation scheme of the FET cycle, which is selected according to the specific situation of the patient. Currently, the natural cycle, artificial cycle, and stimulation cycle are often used. For patients with regular menstruation and normal ovulation, natural cycles are adopted. Artificial cycles were used for patients with anovulation, luteal insufficiency, and a thin endometrium. Stimulation cycles are used for patients with follicular dysplasia, ovulation disorders, polycystic ovarian syndrome (PCOS), or contraindications to estrogen use.

#### Evaluation of pregnancy outcome

A serum β-hCG level ≥50 IU/L on day 14 after transfer, along with a gestational sac observed in the intrauterine cavity on day 35 after transfer, indicated clinical pregnancy. According to the American Society for Reproductive Medicine (ASRM), a miscarriage is defined as a termination of pregnancy at <20 weeks of gestation with a fetal weight of less than 500 g. A live birth is defined as a pregnancy reaching 28 weeks of gestation and resulting in the delivery of a live neonate.

#### Methods for calculating clinical indicators

The clinical indicators were determined as follows: Clinical pregnancy rate = count of clinical pregnancy cycles/count of transfer cycles × 100%; live birth rate = count of live birth cycles/count of transfer cycles × 100%; Abortion rate = count of abortion cycles/count of transfer cycles × 100%; and euploidy rate = count of euploid embryos/count of embryos with PGT.

### Statistical analysis

All data were analyzed using SPSS 25.0. Measurement data are indicated as mean ± standard deviation (SD), and continuous variables were analyzed using a *t*-test. Count data are shown as percentages. Chi-square analysis was used to compare the rates between groups. Logistic regression was applied to control for confounding factors. A *p*-value less than 0.05 was considered statistically significant.

## Results

### Comparison of the basic clinical data and the clinical outcomes between the two groups

A total of 10,146 sFET cycles were compared in this study, of which 9,842 were in the good quality group and 304 were in the low-quality group. Female age, male age, and infertility duration in the good-quality group were all significantly lower than those in the low-quality group (*p* < 0.05). In addition, compared with the good-quality group, the clinical pregnancy rate and the live birth rate were significantly lower in the low-quality group (*p* < 0.001). After adjusting for female age, male age, infertility duration, and other potential confounders, the difference maintained statistical significance [adjusted odds ratio (aOR) = 0.32 and 0.35, *p* < 0.001] (**Table 1**).

**Table 1:** Comparison of basic clinical data and clinical outcomes among the two groups.

Basic clinical data and outcomes	Good-quality group (*n* = 9,842)	Low-quality group (*n* = 304)	*p*-value	aOR (95%CI)^a^	a*p-*value^a^
Female age (years)	31.7 ± 4.6	32.9 ± 5.1	<0.001	–	–
Male age (years)	32.5 ± 5.2	33.8 ± 5.8	<0.001	–	–
Female body mass index (kg/m^2^)	24.0 ± 3.4	23.8 ± 3.1	0.35	–	–
Infertility type (%)			0.52	1.05 (0.83–1.33)	0.68
Primary infertility	3,584 (36.4%)	116 (38.2%)			
Secondary infertility	6,258 (63.6%)	188 (61.8%)			
Main cause of infertility (%)			<0.001		
Female subjects	6,082 (61.8%)	227 (74.7%)			
Male subjects	1,575 (16.0%)	33 (11.0%)			
Mixed	2,126 (21.6%)	41 (13.2%)			
Other	59 (0.6%)	3 (1.1%)			
Infertility duration (years)	3.2 ± 2.6	3.7 ± 3.4	0.001	1.04 (1.01–1.08)	0.02
Endometrial preparation method			0.032		
Artificial cycle	4,652 (47.3%)	328 (50.1%)		1.00 (reference)	–
Natural cycle	3,997 (40.6%)	271 (41.4%)		0.98 (0.82–1.17)	0.82
Stimulated cycle	1,193 (12.1%)	56 (8.5%)		0.79 (0.58–1.08)	0.14
Clinical pregnancy rate	5,721 (58.0%)	83 (27.3%)	<0.001	0.32 (0.25–0.41)	<0.001
Miscarriage rate	1,059 (10.7%)	20 (6.6%)	0.021	0.72 (0.45–1.16)	0.18
Live birth rate	4,662 (47.2%)	63 (20.7%)	<0.001	0.35 (0.26–0.46)	<0.001

a Adjusted for female age, male age, infertility duration, infertility type, and endometrial preparation method.

### Effect of age on the pregnancy outcomes from sFET in the good-quality and low-quality groups

Patients were divided into three different age groups: <35 years old (the <35 group), 35–39 years old (the 35–39 group), and ≥40 years old (the ≥40 group). The effects of age on the pregnancy outcomes from sFET in the good-quality and low-quality groups were compared.

**Figure 1:**
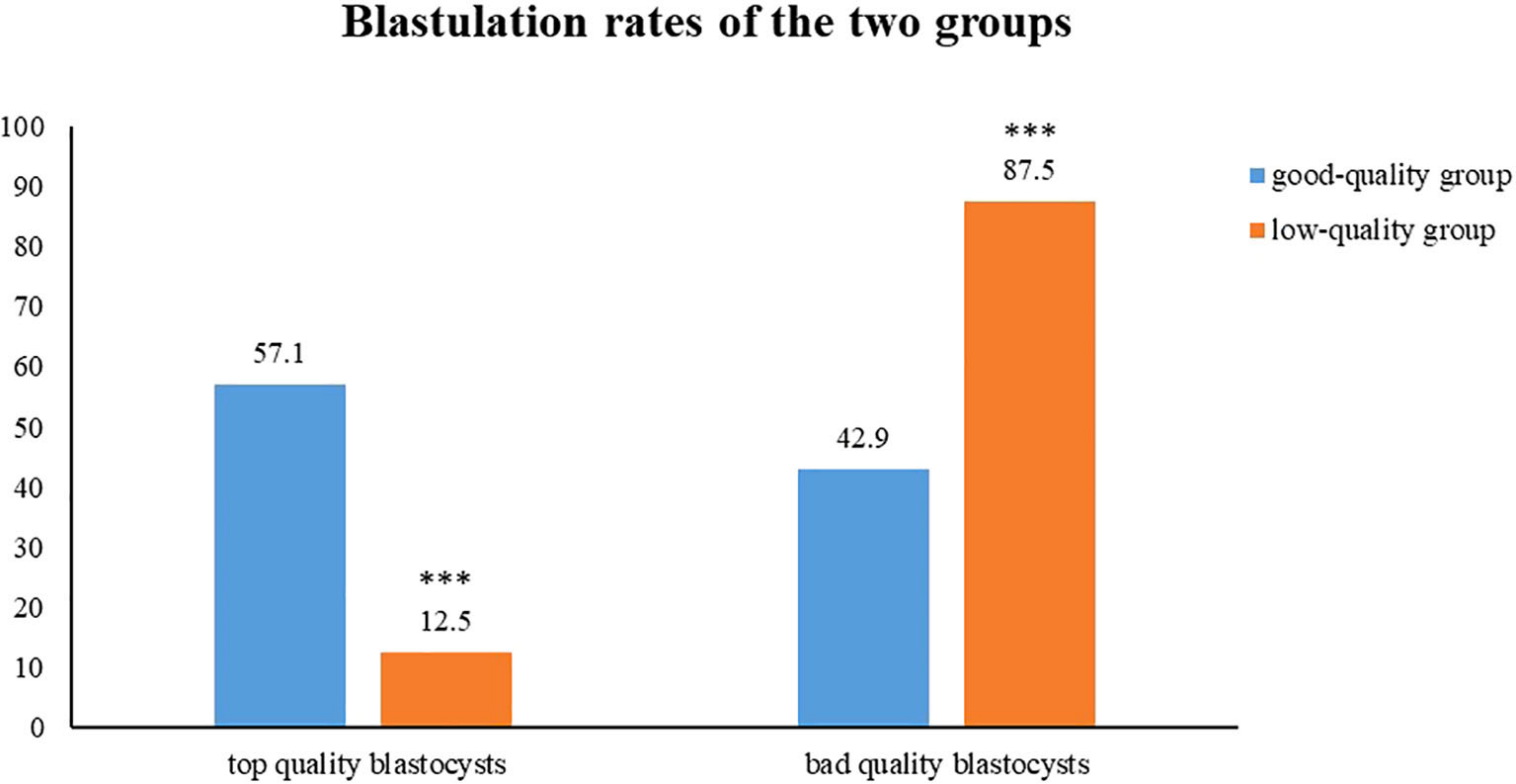
The comparison of the proportions of high-quality blastocysts and low-quality blastocysts between the two groups. Top quality blastocysts shows 57.1% in the good-quality group (blue) and 12.5% for the low-quality group (orange). Bad quality blastocysts shows 42.9% for good-quality and 87.5% for low-quality.

A comparative analysis of the blastocyst quality scores between the two groups was performed. The analysis revealed a significantly higher proportion of top-quality blastocysts in the good-quality group compared with the low-quality group (57.1% *vs*. 12.5%, *p* < 0.01). Moreover, the rate of bad-quality blastocysts in the low-quality group was higher than that of the good-quality group (42.9% *vs*. 87.5%, *p* < 0.01) (**Figure 1**).

In the same age group, the clinical pregnancy rate and the live birth rate of the good quality group were higher than those in the low-quality group, and the difference between the <35 group and the 35–39 group was statistically significant (*p* < 0.05) (**Table 2**).

**Table 2:** Comparison of pregnancy outcomes between good-quality and low-quality groups within the same age range.

Group	Good-quality group (*n* = 9,842)	Low-quality group (*n* = 304)	*p*-value
<35 years			
ET cycles	7,409	205	
Clinical pregnancy rate	4,548 (61.4%)	55 (26.8%)	0.000
Miscarriage rate	697 (9.4%)	9 (4.4%)	<0.001
Live birth rate	3,829 (38.9%)	46 (22.4%)	0.000
35–39 years			
ET cycles	1,809	64	
Clinical pregnancy rate	957 (52.9%)	20 (31.3%)	0.000
Miscarriage rate	259 (14.3%)	7 (10.9%)	0.416
Live birth rate	689 (38.1%)	13 (20.3%)	0.000
≥40 years			
ET cycles	624	35	
Clinical pregnancy rate	216 (34.6%)	8 (22.9%)	0.153
Miscarriage rate	70 (11.2%)	3 (8.6%)	0.591
Live birth rate	144 (23.1%)	5 (14.3%)	0.226

*ET*, embryo transfer.

Multivariate regression analysis was performed to control for confounding factors (embryo quality and age). After adjusting for embryo quality, the clinical pregnancy rate and the live birth rate in the <35 group were significantly higher than those in the ≥40 group (OR = 3.02 and 3.56, *p* < 0.001). The pregnancy and live birth rates in the 35–39 group were still higher than those in the ≥40 group, but the odds ratios decreased (OR = 1.89 and 1.84, *p* < 0.001). Embryo quality significantly affected the clinical pregnancy rate and the live birth rate (*p* < 0.001) (**Table 3**; **Supplementary Table S1**).

**Table 3:** Comparison of pregnancy outcomes of blastocyst transfer from the good-quality group and the low-quality group in different age groups.

Variable	Clinical pregnancy rate: aOR (95%CI)	*p-*value	Live birth rate: aOR (95%CI)	*p*-value
Age group				
<35 years	3.15 (2.67–3.72)	<0.001	3.62 (2.99–4.38)	<0.001
35–39 years	1.91 (1.58–2.31)	<0.001	1.87 (1.51–2.32)	<0.001
Embryo quality				
Good-quality (*vs*. low-quality)	2.92 (2.24–3.80)	<0.001	2.98 (2.23–3.98)	<0.001

Adjusted for embryo quality. Reference group: ≥40 years

*aOR*, adjusted odds ratio.

### Euploidy rates of the good-quality group and the low-quality group in the PGT cycles

The results of the 650 cycles of sFET with PGT from March 2016 to September 2022 were included. The euploidy rate of the blastocysts from the low-quality group was higher than that of the good-quality group; however, the difference was not statistically significant (*p* = 0.561) (**Table 4**).

**Table 4:** Comparison of the euploidy rates in the good-quality group and the low-quality group in pre-implantation genetic testing (PGT) cycles.

	High-quality group (*n* = 635)	Low-quality group (*n* = 15)	*p*-value
Euploidy rate			0.561
No	73 (11.5%)	1 (6.7%)	
Yes	562 (88.5%)	14 (93.3%)	

### Basic clinical data and outcomes of the sFET cycles derived from low-quality embryos in the PGT and non-PGT groups

There were 15 and 413 sFET cycles derived from low-quality embryos in the PGT and non-PGT groups, respectively. Compared with the non-PGT group, the clinical pregnancy rate and the live birth rate in the PGT group were higher; moreover, the miscarriage rate in the PGT group was lower. No significant differences were found (*p* > 0.05) (**Table 5**).

**Table 5:** Basic clinical data and outcomes of single blastocyst frozen–thawed transfer (sFET) cycles derived from low-quality embryos of pre-implantation genetic testing (PGT) and non-PGT.

Basic clinical data and outcomes	PGT cycles (*n* = 15)	Non-PGT cycles (*n* = 413)	*p*-value
Female age (years)	31.1 ± 4.7	32.3 ± 5.0	0.27
Male age (years)	33.5 ± 5.5	33.3 ± 5.5	0.58
Female body mass index (kg/m^2^)	23.9 ± 3.1	23.7 ± 3.0	0.87
Infertility type (%)			0.55
Primary infertility	7 (46.7%)	161 (39.0%)	
Secondary infertility	8 (53.3%)	252 (61.0%)	
Infertility duration (years)	3.0 ± 2.6	3.7 ± 3.2	0.41
Main cause of infertility (%)			0.39
Female subjects	13 (86.7%)	314 (76.5%)	
Male subjects	1 (6.7%)	37 (8.9%)	
Mixed	1 (6.7%)	62 (15.0%)	
Endometrial preparation method			0.05
Artificial cycle	11 (73.3%)	202 (48.9%)	
Natural cycle	3 (20.0%)	184 (44.6%)	
Stimulated cycle	1 (6.7%)	27 (6.5%)	
Clinical pregnancy rate	6 (40.0%)	121 (29.3%)	0.37
Miscarriage rate	0 (0%)	26 (6.3%)	0.28
Live birth rate	6 (40.0%)	91 (22.0%)	0.10

## Discussion

Morphological assessment continues to serve as the primary method for evaluation of embryo development potential. However, it has several limitations. Morphological scoring relies on visual assessment, which can be subjective. Additionally, morphology does not detect chromosomal abnormalities (aneuploidy), which are a major cause of implantation failure and miscarriage (10). Furthermore, high morphological scores do not always correlate with successful implantation or live birth (11, 12). In addition, many embryos may receive the same high score, making it difficult to choose the single best one for transfer.

In assisted reproductive technology, embryos with poor morphological scores are generally considered to have lower developmental potential. However, emerging research has demonstrated that some low-morphological-score embryos may undergo self-repair mechanisms to restore normal development and even achieve successful pregnancies (13, 14). Embryos with significant fragmentation (>25%) can undergo intrinsic repair processes during blastocyst development. These self-repair mechanisms, including lysosomal degradation of cellular fragments, enable certain fragmented embryos to achieve morphological normalization and to develop into viable blastocysts for transfer (15). It has been demonstrated that, during the development of D3 embryos into blastocysts, with the activation of the embryo genome, embryos with genetic and metabolic defects will be naturally eliminated, and a portion of these embryos appear to be able to repair themselves and eventually develop into blastocysts (16–18). Furthermore, with the development of blastocyst culturing and freezing technology, low-quality embryos will still have the potential to develop into blastocysts, even into high-quality blastocysts when cultured *in vitro* (18). Studies have shown that the blastocysts derived from low-quality embryos have the potential to deliver healthy babies successfully after freezing and thawing (3). In this study, it was found that the clinical pregnancy rate and the live birth rate were significantly lower in the low-quality group than in the high-quality group. Although the results revealed that embryo quality is an independent predictor of pregnancy outcomes, live birth rates in patients who underwent freeze–thaw transfer of single blastocysts derived from low-quality D3 embryos accounted for 20.7%, which may be related to the self-repair function of the embryo (19).

Age is another independent factor affecting assisted reproductive technology pregnancy outcomes. In this study, it was found that the parental ages in the low-quality group were significantly higher than those in the high-quality D3 group, suggesting that the advanced age of couples can affect the embryo quality in the cleavage stage. Consistent with a previous study, we found that whether the transplanted blastocysts were from the good-quality or the low-quality D3 group, the clinical pregnancy rate and the live birth rate in the <35-year-old group were highest (20). In the same age group, the live birth rate of the good-quality D3 group was higher than that of the low-quality group. In addition, age is an independent factor affecting live birth rates. With advancing age, the aneuploidy rate of embryos increases by 10%, which is also the main cause of embryo implantation failure and abortion in elderly (≥40 years old) patients during IVF cycles (21, 22).

Aneuploidy is the main cause of spontaneous abortion. Munne et al. reported that the aneuploidy rate of embryos was 63% and that the chromosome aneuploidy rate of embryos in the low-quality group was higher than that of the good-quality group in women aged 35–37 years (23). However, Lee et al. reported that the most obvious association between chromosomes and morphology concerned embryo gender rather than aneuploidy (24). In this study, there was no statistically significant association between the morphological score and the euploidy rate (*p* = 0.561). Nevertheless, the small sample size in the low-quality PGT group limited the robustness of our findings; thus, this analysis should be considered hypothesis-generating rather than conclusive. More studies with larger sample sizes are needed to confirm these findings. Our findings were also consistent with the conclusion of Lee et al., who used a different scoring system. 

For those patients who do not have any embryos, low-quality embryos can be cultured into blastocysts, which can give them a chance for transfer and even a successful pregnancy. Moreover, we compared the clinical outcomes in PGT and non-PGT cycles of the blastocysts from the low-quality group. It was found that the clinical pregnancy rate and the live birth rate of the blastocysts from the low-quality group in the PGT cycles were higher than those in the non-PGT cycles. These results indicate that biopsy and PGT significantly enhance blastocyst utilization efficiency in the low-quality group while reducing unnecessary embryo transfers.

This study did not evaluate neonatal outcomes (such as birth defects and preterm birth), which is a significant limitation. Although there is controversy in the existing literature (25) regarding the association between embryo quality and perinatal outcomes, low-quality D3 embryos may be subjected to additional stress when cultured *in vitro* to the blastocyst stage, theoretically increasing the risk (26, 27). Future research should verify this hypothesis through the design of birth cohort studies (such as follow-up until infancy).

In summary, although embryos with development potential can be screened by further cultivation to blastocysts, the aneuploidy rate of blastocysts from low-quality embryos is still high. Thus, for infertile couples without good-quality D3 embryos, blastocyst culture of low-quality embryos and PGT can be performed to obtain euploid blastocysts, which can improve the clinical pregnancy and live birth rates.

## Data Availability

The raw data supporting the conclusions of this article will be made available by the authors without undue reservation.
