# Supplementary material for: Clinical outcomes of frozen–thawed single blastocyst transfer derived from low-quality day 3 embryos: A retrospective cohort study
Source: Front Endocrinol (Lausanne). 2025 Jul 28;16:1583779. doi: 10.3389/fendo.2025.1583779 (PMC12336024; doi:10.3389/fendo.2025.1583779)
Supplement: Supplementary file 1 [file Table1.docx]

**Table S1 Comparison of pregnancy outcomes of blastocyst transfer from good-quality group and low-quality group in the different age groups**

|  | cycles | clinical pregnancy rate | miscarriage rate | live birth rate |
| --- | --- | --- | --- | --- |
| **＜35 years** | 7614 | 4603 (60.5%) | 728 (9.6%) | 3875 (50.9%) |
| good-quality group | 7409 | 4548 (61.4%) | 719 (9.7%) | 3829 (38.9%) |
| low-quality group | 205 | 55(26.8%) | 9 (4.4%) | 46 (22.4%) |
| **35-39 years** | 1873 | 669 (49.2%) | 196 (14.4%) | 473 (34.8%) |
| good-quality group | 1809 | 957 (52.9%) | 268 (14.8%) | 689 (38.1%) |
| low-quality group | 64 | 20 (31.3%) | 7 (10.9%) | 13 (20.3%) |
| **≥40 years** | 659 | 224 (34.0%) | 75 (11.4%) | 149 (22.6%) |
| good-quality group | 624 | 216 (34.6%) | 72 (11.5%) | 144 (23.1%) |
| low-quality group | 35 | 8 (22.9%) | 3 (8.6%) | 5 (14.3%) |
